# Mass spectrometry analysis of transthyretin (TTR) post-translational modifications (PTMs) in hereditary ATTR: a case-control Spanish experience

**DOI:** 10.1186/1750-1172-10-S1-P53

**Published:** 2015-11-02

**Authors:** Marta Vilà-Rico, Sebastián Azorín Contesse, José E Barcena Llona, Ricardo Rojas-García, Fernando Martínez Valle, Antoni Planas, Francesc Canals, Josep M Campistol

**Affiliations:** 1 Institut Químic de Sarrià, Universitat Ramon Llull, Laboratory of Biochemistry, 08017, Barcelona, Spain; 2 Hospital clínic de Barcelona, Amyloidosis and monoclonal gammopathies’ unit (UDAM) - Nephrology and transplant unit (SNiTR), 08036, Barcelona, Spain; 3 Cruces university hospital, Multiple sclerosis and demyelinating diseases unit, Neurology service, Department of neurosciences, 48903, Biscay, Spain; 4 Hospital de la Santa Creu i Sant Pau, Neuromuscular disease unit, Neurology service, 08025, Barcelona, Spain; 5 Vall d'Hebron University Hospital, Internal medicine department I, 08035, Barcelona, Spain; 6 Vall d'Hebron Institute of Oncology, Proteomics laboratory, 08035, Barcelona, Spain

## Background

Transthyretin (TTR) is an amyloidogenic tetrameric protein, present in human plasma, associated with several familial amyloidoses. Variability of TTR is not only due to point mutations in the encoding gene but also to post-translational modifications (PTMs) at Cys10, being the most common PTMs the S-sulfonation, S-glycinylcysteinylation, S-cysteinylation and S-glutathionylation. It is thought that PTMs at Cys10 may play an important biological role in the onset and pathological process of the amyloidosis. Recently we reported the development of a methodology for quantification of PTMs in serum samples, as well as for the determination of serum TTR levels, from healthy (wt-TTR) and ATTR V30M individuals which involves an enrichment step by immunoprecipitation followed by mass spectrometry analysis of (i) the intact TTR protein and (ii) targeted LC-MS analysis of peptides carrying the PTMs of interest (M Vilà-Rico et al. Analysis of post-translational modifications in human transthyretin associated with familial amyloidotic polyneuropathy by targeted LC-MS and intact protein MS. Journal of Proteomics (2015) in press). Analysis of serum samples by the combination of the two methods affords complementary information on the relative and absolute amounts of the selected TTR PTM forms.

## Methods

We aimed at describing the applicability of our mass spectrometry methodology among healthy controls and V30M-TTR patients (cases) at different disease stages followed at our institution and other three spanish health facilities. Inclusion criteriae for cases consisted of a positive genetic testing for V30M-TTR status and a signed ethics’ committee approved informed consent. Mass spectrometric analysis was performed as detailed in our previouis work.

## Results

A total of 50 healthy controls and 29 patients were included after signing the informed consent. Demographic and clinical characteristics were gathered and correlated to the proteomic analysis profile according to our validated methodology. Significant differences were found for total TTR as well as for specific TTR Cys-10 PTMs between the different V30M ATTR stages as well as between controls and symptomatic patients.

## Conclusion

Quantification of wt:V30M TTR ratio and quantification of Cys-10 PTM.

Isoforms is a feasible, reproducible and robust method by intact TTR and targeted LC-MS in the TTR V30M population. Significant differences for wt:V30M TTR ratio as well as for some specific PTMs have been found in a small cohort of V30M-TTR patients at different ATTR stages. These results need to be confirmed in a bigger cohort of patients with ATTR.

